# Entire Wheat Flour

**Published:** 1884-10

**Authors:** 


### ENTIRE WHEAT FLOUR.

THERE has never until recently been known any possible means or way to make all of the wheat fine; hence the millers have given us nothing but "the beautiful white flour" from which the best or nutritious part of the wheat is eliminated, or the so-called "Graham" flour – a name, title or brand which causes a multitude of sins. Most of the "Graham" flour sold in this country is nothing but a mixture of the lowest grades of white flour with bran. No physician who is posted on cereal foods, and knows the merits of the entire wheat flour, will advise any one to eat "Graham" flour; while every physician in this country and England who has seen and knows what it is, uses and recommends the entire wheat flour, which fact is explained by a short statement of the way it is made, viz.:

The wheat is first cleaned in the usual way, then it goes to a machine which takes off the skin or husk; then it is reduced, not ground, by the regular roller process (except purifiers); then after the separation by bolting of the bran from the white flour, the bran is reduced by special machinery; then by a system of spouting, the bean and white flour is brought together and mixed in exactly the same proportion that existed in the berry.

This flour is not only much more nutritious than any other, but will assimilate with the weakest stomach, because it is fine and contains all the gluten and phosphates that are in wheat; which can be said of no other flour in the world. It is cheaper than any other, because it makes so much more bread—which is explained by the theory of porosity, which theory is demonstrated by the fact known to every baker or bread-maker, that a good Minnesota patent flour will make twenty-five per cent. more bread than the best grade of Graham flour or wheat meal; and the entire wheat flour makes twenty-five to thirty per cent more bread than the Minnesota patent flour. The roller process, which makes the best and highest-priced white flour in the world, was a great and glorious advance toward the right kind of flour for the people; but now we have a still greater advance in the art of milling, which gives the people the wheat as it grows, or a fine flour of the entire wheat except the skin which is not a food, and which, alone, cattle will not eat. The long continued use of flour usually sold as Graham flour, is positively dangerous to health. Dyspepsia is always made worse by its use. A large proportion of the bran found in such flour is mixed with the silicate coating of wheat, and it cuts the lining of the stomach like pieces of glass. Entire wheat flour is quite another thing. We prove our faith in it by using it in preference to any other.

